# Bleomycin Revisited: A Direct Comparison of the Intratracheal Micro-Spraying and the Oropharyngeal Aspiration Routes of Bleomycin Administration in Mice

**DOI:** 10.3389/fmed.2018.00269

**Published:** 2018-09-24

**Authors:** Ilianna Barbayianni, Ioanna Ninou, Argyrios Tzouvelekis, Vassilis Aidinis

**Affiliations:** Division of Immunology, Biomedical Sciences Research Center Alexander Fleming, Athens, Greece

**Keywords:** pulmonary fibrosis, animal model, bleomycin (BLM), intratracheal (IT), oropharyngeal (OA)

## Abstract

Idiopathic Pulmonary Fibrosis (IPF) is a fatal disease characterized by exuberant deposition of extracellular matrix components, deterioration of lung architecture and impairment of lung functions. Its etiopathogenesis remains incompletely understood, as reflected in the lack of an appropriate therapy. Modeling the human disease in mice via the administration of bleomycin (BLM), despite the inherent limitations, has provided valuable insights into the underlying pathogenetic mechanisms, and has been instrumental for the development and validation of new pharmacologic interventions. Here we have directly compared the, most widely used, intratracheal (IT) route of administration with oropharyngeal aspiration (OA). Our results suggest that the OA route of BLM-administration can be used as a safe and effective alternative, minimizing peri-operative and experimental mortality, while preserving a solid fibrotic profile, as assessed with a plethora of standardized readout assays.

## Introduction

Modelinghuman diseases in mice, despite the inherent limitations, has provided valuable insights into the underlying pathogenetic mechanisms, and has been instrumental for the development and validation of new pharmacologic interventions. In the context of Idiopathic Pulmonary Fibrosis (IPF), a fatal disease characterized by exuberant deposition of extracellular matrix components, deterioration of lung architecture and impairment of lung functions (1), the most widely used experimental model is induced by the administration of bleomycin in C57Bl6/J mice (2–4).

Bleomycin (BLM), a mixture of glycopeptides isolated from *Streptomyces verticillus*, is an anti-neoplastic/antibiotic drug for the treatment of some forms of cancer (5). It acts through DNA fragmentation, an activity modulated by many factors in different cell types including chromatin structure and DNA repair machinery, as well as antioxidant and metabolic enzymes (5). The lack of BLM hydrolase in pulmonary epithelial cells is thought to be the main reason for the observed toxicity of BLM in the lung, resulting in the development of pulmonary fibrosis as a side effect in treated cancer patients. The observed toxicity in human patients was soon translated to an animal model (6), serving the scientific community ever since. The model is characterized by alveolar epithelial cell death and the secretion of pro-inflammatory and pro-fibrotic factors, leading to fibroblast activation and collagen deposition, reproducing some, but not all, of the key features of the human disease (3, 4). The ensuing inflammation, the lack of alveolar epithelial hyperplasia and the quick resolution, the main differences with the human disease and the major drawbacks of the BLM model, can be circumvented, in part, though the repetitive administration of BLM (7).

In order to mimic the human exposure, BLM was initially administered systemically, via intravenous or intraperitoneal injections, resulting, as in humans, to the subpleural development of fibrotic lesions (4). However, intratracheal (IT) administration of a single BLM dose, resulting in bronchiolocentric fibrotic patches, has become the method of choice (3). Furthermore, the IT delivery of BLM through a microspray aerosolizer (8) has been shown to yield more reproducible results and more homogenous distribution of fibrotic lesions than IT instillation/injection (9). Following the example of other drugs and agents (10–13), an alternative way of administering BLM in the trachea, namely oropharyngeal aspiration (OA), has been recently introduced (14, 15). However, the IT and OA routes of BLM administration have not been directly compared (3). As shown here, OA of 0.8 U/Kg BLM results in similar fibrotic responses as with 3.2 U/Kg when administered IT. OA administration reduced both the peri-operative mortality, due to the ease and speed of procedures, while the lower BLM dose employed also reduced experimental mortality.

## Materials and methods

### Mice

Mice were bred under SPF conditions at the local animal facility at “20–22°C, 55 ± 5% humidity, and a 12-h light-dark cycle; water and food were given *ad libitum*” (17). All experimentation in mice was in line with the ARRIVE guidelines and has been approved by the Veterinary service and Fishery Department of the local governmental prefecture, following the approval by the Institutional Animal Ethical Committee (IAEC; #985) of BSRC Alexander Fleming.

Pulmonary fibrosis was induced through the administration of BLM (Nippon Kayaku) to anesthetized (IP ketamine/xylazine/atropine, 100/10/0.05 mg/kg, respectively) mice. The intratracheal (IT) route was applied essentially as previously published (17), and as described in the online Supplementary Materials and Methods. Briefly, a MicroSprayer aerosolizer attached to a high-pressure syringe was inserted from the mouth to the carina (trachea's bifurcation) and BLM (0.08 U/mouse), or saline, was sprayed directly into the lungs of mice). The oropharyngeal (OA) route was applied as follows: the tongue of the mice was carefully pulled out using blunt forceps while the mouse's neck and thorax were stabilized on a plastic wall through a rubber band in order for the neck to be minimally stretched. The latter permitted the visualization of the trachea through a laryngoscope and a fiber- optic device. BLM (in a final volume of 50 μl) was then delivered as liquid in the oropharyngeal cavity, with a blunt ended conventional pipette tip. At the same time, the nares were blocked by a tong to prevent obligate nasal breathing and force BLM inhalation. Once BLM was administered (IT or OA), mice were placed on an electrical heating blanket to ensure speedy recovery from anesthesia and to avoid hypothermia.

Bronchoalveolar Lavage Fluid (BALF) collection and analysis, lung histopathological analysis and Quantitative RT-PCR analysis were performed with standardized protocols, as previously published (16) and as described in the on-line Supplementary Materials and Methods.

Respiratory mechanics were analyzed with the FlexiVent ventilator system **(**SCIREQ**)** following manufacturer instructions, as previously published (17) and as described in the on-line Supplementary Materials and Methods.

### Statistical analysis

Statistical significance was assessed with unpaired Student's *t*-test in comparison with control values (GraphPad Prism 6). Data are presented as means (±SEM); *p* < 0.05 (^*^) was considered significant.

## Results and discussion

Beyond the route of administration, the severity of BLM effects highly depends on the precise genetic background of mice (i.e., C57Bl6 J vs. N, further differing between vendors), the local genetic drift of the colony and the health status of the corresponding animal house. As a result, a wide range of BLM concentrations have been employed to induce pulmonary fibrosis in mice (2, 4). As there are only a few published protocols on the OA route of BLM administration, we first tested four different BLM doses administered by OA. The starting concentration was 3.2 U/Kg, the concentration used locally for the IT route, which has been chosen after extensive testing over the years to establish a reproducible phenotype with minimal lethality. Administering 3.2 U/Kg BLM via OA, as well as to a lesser extend 1.6 U/Kg, resulted in significant mortality rates (Figure S1A), so these concentrations were discontinued. On the contrary, doses of 0.4 and 0.8 U/Kg were well tolerable, while the dose of 0.8 U/Kg produced statistically significant increases in all established diseases indices (Figures S1B–H) with minimal mortality and was thus selected for the direct comparison of IT and OA routes.

**Figure 1:**
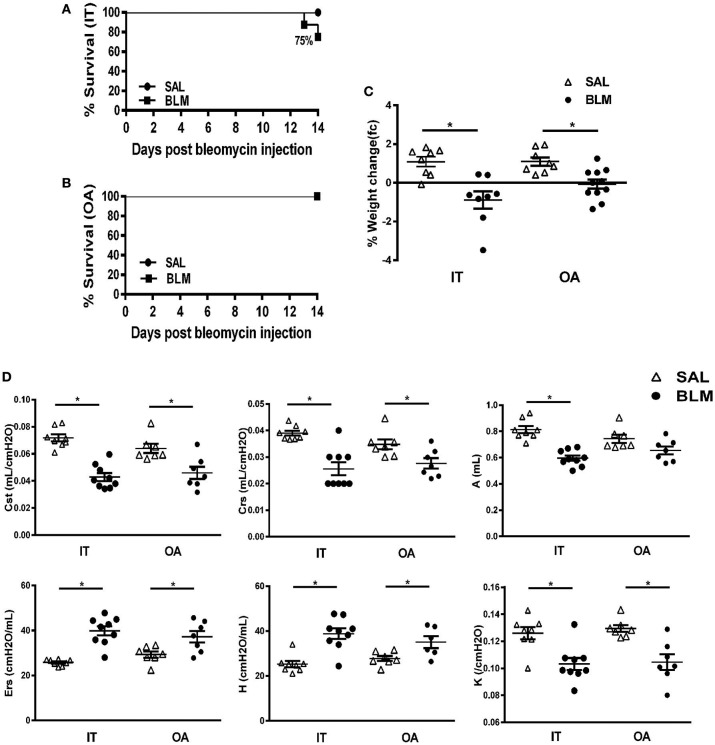
Effects of the intratracheal microspraying (IT) or oropharyngeal aspiration (OA) routes of bleomycin (BLM) administration on mortality, weight loss and functional respiratory mechanics. 8–12 weeks-old C57BL6/J mice were challenged with BLM delivered via the IT or OA routes (at doses of 3.2 and 0.8 U/kg, respectively) and were sacrificed 14 days later. Data from two independent experiments are presented as scatter plots with horizontal bars representing mean levels (±SEM). Statistical significance was assessed with unpaired Student's *t*-test in comparison with the relative control values; ^*^p < 0.05 was considered statistically significant. **(A,B)** Kaplan-Meier plot using 14-days survival data from mice treated with BLM delivered either through IT or OA route, respectively. **(C)** Both OT and IA-treated mice demonstrated marked weight loss compared to saline-treated animals 14 days following BLM-challenge. **(D)** *In-vivo* respiratory mechanics following challenge with BLM. OA administration exerted similar to IT administration significant functional impairment on respiratory mechanics compared to saline-treated controls, as assessed by: mean static lung compliance (*Cst*), mean respirator system compliance (*Crs*), mean total lung capacity (*A*), mean respiratory system elastance (*Ers*), mean tissue elastance (*H*) and the curvature of the upper portion of the deflation limp of the pressure volume (PV) curve (*K*).

Pulmonary fibrosis was induced by IT or OA administration of BLM (at 3.2 and 0.8 U/Kg, respectively) in both male and female, 8–12 weeks old, C57Bl6/J mice. No sex effect was observed in any readout assays, so all following experimental results concern cumulative data, of randomly assigned, sex and age matched groups of littermate mice. No statistically significant difference on overall mouse survival was found between IT and OA BLM administrations (Figures 1A,B); however, at these doses, no mice died upon OA administration, most likely due to the lower BLM dose employed. Similarly, both routes of BLM administration, as compared to saline-treated animals, resulted in significant weight loss (Figure 1C), one of the traditional indicators of BLM-induced injury. However, IT administration (of BLM or saline) always resulted in peri-operative mortality (data not shown), a feature not usually reported (or even recorded), as it concerns almost exclusively handling, skill and chance. Nevertheless, OA administration is deemed advantageous on overall experimental mice survival, with both practical and ethical benefits. Moreover, the experimental OA procedure is much easier and faster, as described in detail in Supplementary Materials and Methods, maximizing productivity and reproducibility, while it requires much less training.

Fourteen days post BLM (or saline) administration mice were sedated, tracheotomized and connected to a mechanical ventilator to evaluate forced-oscillation lung mechanics (Figure 1D). OA administration exerted similar to IT administration functional impairment on respiratory mechanics compared to saline-treated controls, as assessed by significant reductions in: (1) static lung compliance (*Cst*), (2) respiratory system compliance (*Crs*), and (3) total lung capacity (*A*), as well as increases in: (4) respiratory system elastance (*Ers*), (5) tissue elastance (*H*), (6) curvature of the upper portion of the deflation limb of the PV curve (*K*), (Figure 1D). Both static and dynamic lung compliance as well as elastance and total (inspiratory) lung capacity were found to be reliable indices of fibrotic lung injury, as recently suggested (18), well correlating with the Ashcroft score (Table S1). Therefore, and as the method is the most relevant to clinical measurements in human patients, not requiring additional mouse numbers, it is thus proposed as a valuable surrogate analysis of BLM-induced pulmonary fibrosis.

**Figure 2:**
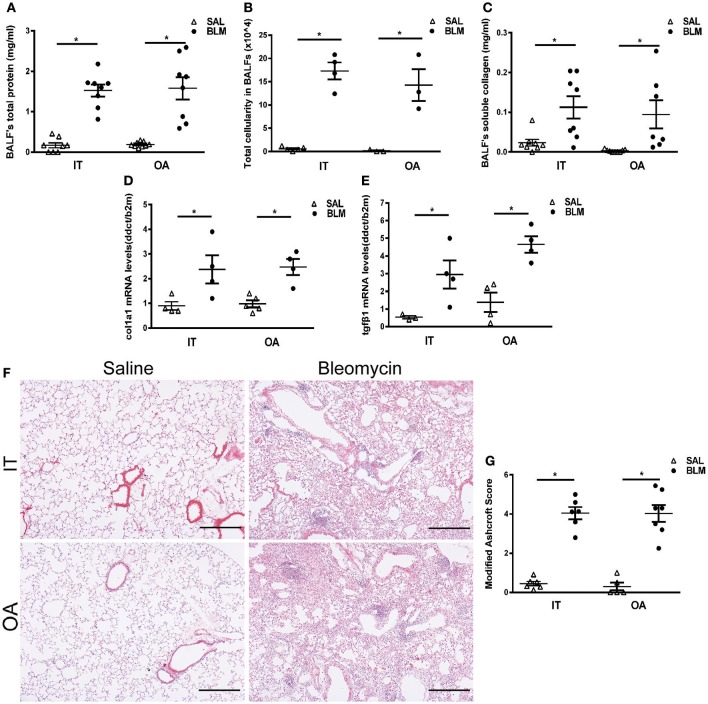
Biochemical and histological analysis of injured lungs following intratracheal microspraying (IT) or oropharyngeal aspiration (OA) bleomycin (BLM) administration. 8–12 weeks-old C57BL6/J mice were challenged with BLM delivered via the IT or OA routes (at doses of 3.2 and 0.8 U/kg, respectively) and were sacrificed 14 days later. Data from two independent experiments are presented as scatter plots with horizontal bars representing mean levels (±SEM). Statistical significance was assessed with unpaired Student's *t*-test in comparison with the relative control values; ^*^p < 0.05 was considered statistically significant. **(A)** Increased total protein levels in BALF were observed with both routes of BLM delivery compared to saline-treated controls. **(B)** Both routes of delivery (IT and OA) produced significantly increases in bronchoalveolar lavage fluid (BALF) total cellularity compared to saline-treated animals. **(C)** Lung collagen was assessed by measuring BALF soluble collagen content with sirius red. Both routes of BLM administration were associated with substantial increases in BALF soluble collagen content compared to saline-treated animals. **(D,E)** Quantitative RT-PCR analysis of the *Col1a1* and *Tgfb* mRNA levels in whole mouse lungs challenged with BLM either through IT or OA route of delivery and saline-treated animals. Values were normalized to the expression values of *b2m*. **(F)** Representative H&E-stained lung sections. Scale bars 100 μm. **(G)** Quantitative analysis of histological changes and extent of fibrosis was performed by the modified Ashcroft score. Data represent mean scores obtained from two independent blind reviewers.

The route of BLM administration did not have an effect in BLM-induced vascular leak, as indicated by the total protein levels in the corresponding bronchoalveolar fluids (BALFs) (Figure 2A). Similarly, no differences were detected in the total number of inflammatory cells in BALFs, upon measuring trypan blue stained cells in a hematocytometer (Figure 2B). Moreover, no qualitative differences in inflammation was detected either at this final endpoint with FACS analysis (data not shown). BALFs were also analyzed for soluble collagen content with sirius red, as an indirect indicator of tissue fibrosis. Again, no difference was noted upon the differential administration route of BLM (Figure 2C). It should be noted that measuring the hydroxyproline content of lung tissue is the most accurate method of determining lung collagen content and, as such, has been recommended as the optimal primary endpoint for fibrosis assessment (3). However, this technique requires at least half the lung, thus limiting the number of parallel analyses that can be performed (either in the mRNA or protein or enzymatic activity level accordingly) or requiring additional mouse numbers, that would still not allow for direct comparisons or correlations of collagen content with other disease indices. The estimation of collagen levels was complemented with Real Time RT-PCR assessment of Collagen 1a1 mRNA levels, again not revealing any differences between the two routes of BLM administration (Figure 2D). Similarly, no differences in TGF mRNA levels, the major profibrotic factor driving collagen expression and disease development in both mice and human, were noted (Figure 2E).

In line with the BALF assays, and as shown in representative images of H&E stained lung sections, BLM-challenge promoted extensive fibrotic changes and architectural distortion compared to saline-treated animals, irrespectively of the delivery method (Figure 2F); no major differences in the distribution and homogeneity of fibrotic lesions were observed. Moreover, collagen visualization with Sirius red and Mason trichrome staining of lung sections did not reveal any gross differences between the two methods either (Figures S2A,B); a recently reported automated histological image analysis of fibrotic lungs will further allow objective quantification of lung tissue density as a result of the deposited collagen (18). As expected, quantification of fibrosis by two blind reviewers using the Ashcroft score did not reveal any differences upon differential BLM delivery (Figure 2G).

Overall, as prompted by the recent American Thoracic Society workshop report on the use of animal models (3), we have directly compared for the first time the IT and OA routes of BLM-delivery in mice, using a plethora of readout assays including *in vivo* lung function measurements which are highly clinically relevant. Our results suggest that the OA route can be used as a safe and effective alternative route of BLM administration, allowing researchers to easily produce reproducible and robust kinetics of fibrotic lung injury combined with a beneficial safety profile sparing invasive surgical procedures of the IT administration, as well as the systemic effects of higher BLM doses. Moreover, the low BLM concentrations employed will allow drug testing in animals of a much better shape, without compromising a solid fibrotic profile.

## Author contributions

VA designed the study; IB and IN performed all reported experiments; AT co-analyzed the data and wrote the paper, which was edited by VA and was critically commented by all authors.

### Conflict of interest statement

The authors declare that the research was conducted in the absence of any commercial or financial relationships that could be construed as a potential conflict of interest.
