# Supplementary material for: Bleomycin Revisited: A Direct Comparison of the Intratracheal Micro-Spraying and the Oropharyngeal Aspiration Routes of Bleomycin Administration in Mice
Source: Front Med (Lausanne). 2018 Sep 24;5:269. doi: 10.3389/fmed.2018.00269 (PMC6165886; doi:10.3389/fmed.2018.00269)
Supplement: Supplementary file 1 [file Data_Sheet_1.PDF]

Bleomycin revisited: A direct comparison of the intratracheal micro-spraying and the oropharyngeal aspiration routes of bleomycin administration in mice

Ilianna Barbayianni[1†], Ioanna Ninou[1†], Argyrios Tzouvelekis[1] and Vassilis Aidinis[1*]

[1] *Division of Immunology, Biomedical Sciences Research Center Alexander Fleming, Athens, Greece*

†Equal contribution

Supplementary Materials and Methods

Bleomycin (BLM)-induced pulmonary fibrosis

Lung fibrosis was induced in female and male C57BL6/J mice, 8-12 weeks old, by intratracheal (IT) or oropharyngeal (OA) administration of BLM HCL (Nippon Kayaku, 15mg/vial diluted in 0.9% normal saline solution), following the procedures bellow: mouse was anaesthetized using xylazine/ketamine/atropine mixture (10mg/100mg/0,05mg/kg respectively) delivered intraperitoneally (IP). Anesthetic depth was monitored to verify the mouse was asleep, by observing the loss of pedal reflexes (pinch at both foot pads). Then, mouse was stabilized on a plastic support, adjusted to 45-60° angles, by hanging it on the two front upper teeth with a rubber band. The mouth was opened by pushing carefully the jaw to the front using a small metal laryngoscope. Simultaneously, with blunt forceps, the tongue was gently pulled out of the mouth to get a clear view of the trachea. A light source with a flexible fiber-optic was used for trachea visualization. The position of fiber-optic was adjusted in each mouse just below vocal cords to provide the best view of trachea; a crucial step for the whole procedure. The trachea can be easily visualized by the differences in colors in the mouth cavity. It is appears as a small white light spot, while the mouth cavity has an orange shade.

For intratracheal (IT) administration, a MicroSprayer aerosolizer attached to a high-pressure syringe was used to deliver BLM (0.08U/mouse in a final volume of 100ul) directly into the lungs. The MicroSprayer tip was inserted from the mouth to the carina (trachea's bifurcation) and the bleomycin was sprayed. Concerning the oropharyngeal (OA) administration, BLM (0.02U/mouse in a final volume of 50ul) was delivered as liquid in the oropharyngeal cavity, with a blunt ended conventional pipette tip. On the same time, the nares were blocked by a tong to prevent obligate nasal breathing and permit the compel inhalation of the deliverable. Once BLM was administered, the mouse was placed on an electrical heating blanket to help the speedy recovery from anesthesia and avoid the hypothermia phenomenon.

***In vivo* measurements of lung function**

On the FlexiVent system (SCIREQ, Montreal, Canada) a forced oscillation technique (FOT, single and low frequency) and a pressure-volume loop (PV) perturbation were performed in tracheotomized mice to produce measurements of respiratory mechanics [1]. In the single FOT, total respiratory system (lung, chest wall and airways) elastance (E_{rs}) and dynamic compliance (C_{rs}) were obtained. E_{rs} shows the elastic stiffness of the respiratory system and C_{rs} describes the easiness of respiratory system extension. The low FOT technique distinguished airway and tissue mechanics; elastance (H) of lung tissue was calculated. The PV loop perturbation generated the quasi-static mechanical properties of the respiratory system; vital (total) lung capacity (A) which is an estimation of inspiratory capacity, form of deflating PV loop (K) and the intrisinic elastic properties defined as static compliance (C_{st}). The average of three acceptable measurements (coefficient of determination (COD)>0.90) was presented.

Bronchoalveolar Lavage Fluid (BALF) collection and analysis

BALF was obtained by lavaging the airways with 3ml of 0.9% sterile sodium chloride (three times, 1ml each). After centrifugation at 100g for 10 min, BALFs were stored at -80°C for protein and collagen content determination, while cell pellets were stained with 0.4% Trypan Blue solution and used for total cell count with a hematocytometer, or used for FACS analysis.

Total protein levels were assessed with the Bradford assay for protein according to the instructions by the manufacturer (Bio-Rad, Hercules, CA, USA). Absorbance values were converted in mg/ml using a bovine serum albumin standard curve (BSA 0–2 $mg \cdot mL^{-1}$). Quantification of soluble collagen was performed using the Sirius Red assay protocol: 50μl of BALF samples, diluted in 0.5M acetic acid, were incubated for 30min with Sirius Red at RT (direct red 80; 120 $\mu g \cdot mL^{-1}$ in 0.5 M acetic acid). After centrifugation, the absorbance of supernatant was read at 540nm and values were converted in μg/ml according to a standard curve with collagen type I from rat tail (0–500 $\mu g \cdot mL^{-1}$).

Lung histopathology

The right lung tissues were fixed in 10% v/v neutral buffered formalin and embedded in paraffin. 4μm lung sections were prepared and stained with Haematoxylin/eosin (H&E), Masson's trichrome and Sirius Red with the standard protocols. Histopathologic analysis of fibrosis was performed in a blinded fashion by two independent reviewers using the modified Ashcroft score [2] as follows: 0, normal lung; 1, isolated alveolar septa with gentle fibrotic changes; 2, fibrotic changes of alveolar septa with knot-like formation; 3, contiguous fibrotic walls of alveolar septa; 4, single fibrotic masses; 5, confluent fibrotic masses; 6, large contiguous fibrotic masses; 7, air bubbles; 8, fibrous obliteration. The average score from 10 different chosen fields was presented. Lung tissue imaging was performed using a Nikon Eclipse E800 microscope (Nikon Corp., Shinagawa-ku, Japan) attached to a Q Imaging EXI Aqua digital camera, using the Q-Capture Pro 7 software.

RNA Extraction and Real-time RT-PCR Analysis

The left lung lobe was used for total RNA extraction using the Tri Reagent (Molecular Research Center, Inc., USA). Treatment with DNase (RQ1 RNAse-free DNase, Promega, Wis, USA) was performed in accordance to the manufacturer's instructions. The cDNA was synthesized from 1.5 μg total RNA in a 20-μl reaction using M-MLV RT (Promega). Quantitative real-time polymerase chain reaction (Q-RT-PCR) was performed using SoFAst EvaGreen Supermix on a Bio-Rad CFX96 Touch™ Real-Time PCR Detection System (Bio-Rad Laboratories Ltd, CA, USA). Values were normalized to β2-microglobulin (B2M). Primers used, as well as the product size (bp) were as follows: col1a1 (f, 5′-CTACTACCGGGCCGATGATG-3′, r, 5′-CGATCCAGTACTCTCCGCTC-3′, 188bp), tgfb1 (f, 5'-CTC CCG TGG CTT

CTA GTG C-3', r, 5'-GCC TTA GTT TGG ACA GGA TCT G-3', 133bp), B2M (f, 5´-TTCTGGTGCTTGTCTCACTGA-3’, r, 5´-CAGTATGTTCGGCTTCCCATTC-3´, 104bp). The annealing temperature for all primers was 58°C. Relative gene expression was normalized to a value of 1.0 for the unstimulated control group. Fold change was calculated taking the mean of the controls as the baseline.

References

1. McGovern, T.K., et al., *Evaluation of respiratory system mechanics in mice using the forced oscillation technique.* J Vis Exp, 2013(75): p. e50172.
2. Hubner, R.H., et al., *Standardized quantification of pulmonary fibrosis in histological samples.* Biotechniques, 2008. **44**(4): p. 507-11, 514-7.
