# Supplementary material for: Bleomycin Revisited: A Direct Comparison of the Intratracheal Micro-Spraying and the Oropharyngeal Aspiration Routes of Bleomycin Administration in Mice
Source: Front Med (Lausanne). 2018 Sep 24;5:269. doi: 10.3389/fmed.2018.00269 (PMC6165886; doi:10.3389/fmed.2018.00269)
Supplement: Supplementary file 2 [file Data_Sheet_2.PDF]

Table S1. Correlation of the degree of bleomycin-induced pulmonary fibrosis, as quantified with the Ashcroft score, with respiratory functions.

r^2 (p)		**Respiratory functions - FlexiVent**					
		**H**	**A**	**K**	**Cst**	**Ers**	**Crs**
**Ashcroft score**	**IT**	0.78 (*)	0.98 (***)	0.67 (*)	0.96 (***)	0.85 (**)	0.8 (*)
	**OA**	0.65 (***)	0.48 (**)	0.76 (***)	0.76 (***)	0.64 (***)	0.51 (**)

IT: Intratracheal micro-spraying; OA: oropharyngeal aspiration
*r^2: Pearson correlation coefficient; * $p<0.05$, ** $p<0.01$, *** $p< 0.001$*
H: tissue elastance; A: total lung capacity; K: curvature of the upper portion of the deflation limb of the PV curve; Cst: static lung compliance; Ers: respiratory system elastance; Crs: respiratory system compliance.
