# Supplementary figures and images for: Bleomycin Revisited: A Direct Comparison of the Intratracheal Micro-Spraying and the Oropharyngeal Aspiration Routes of Bleomycin Administration in Mice
Source: Front Med (Lausanne). 2018 Sep 24;5:269. doi: 10.3389/fmed.2018.00269 (PMC6165886; doi:10.3389/fmed.2018.00269)

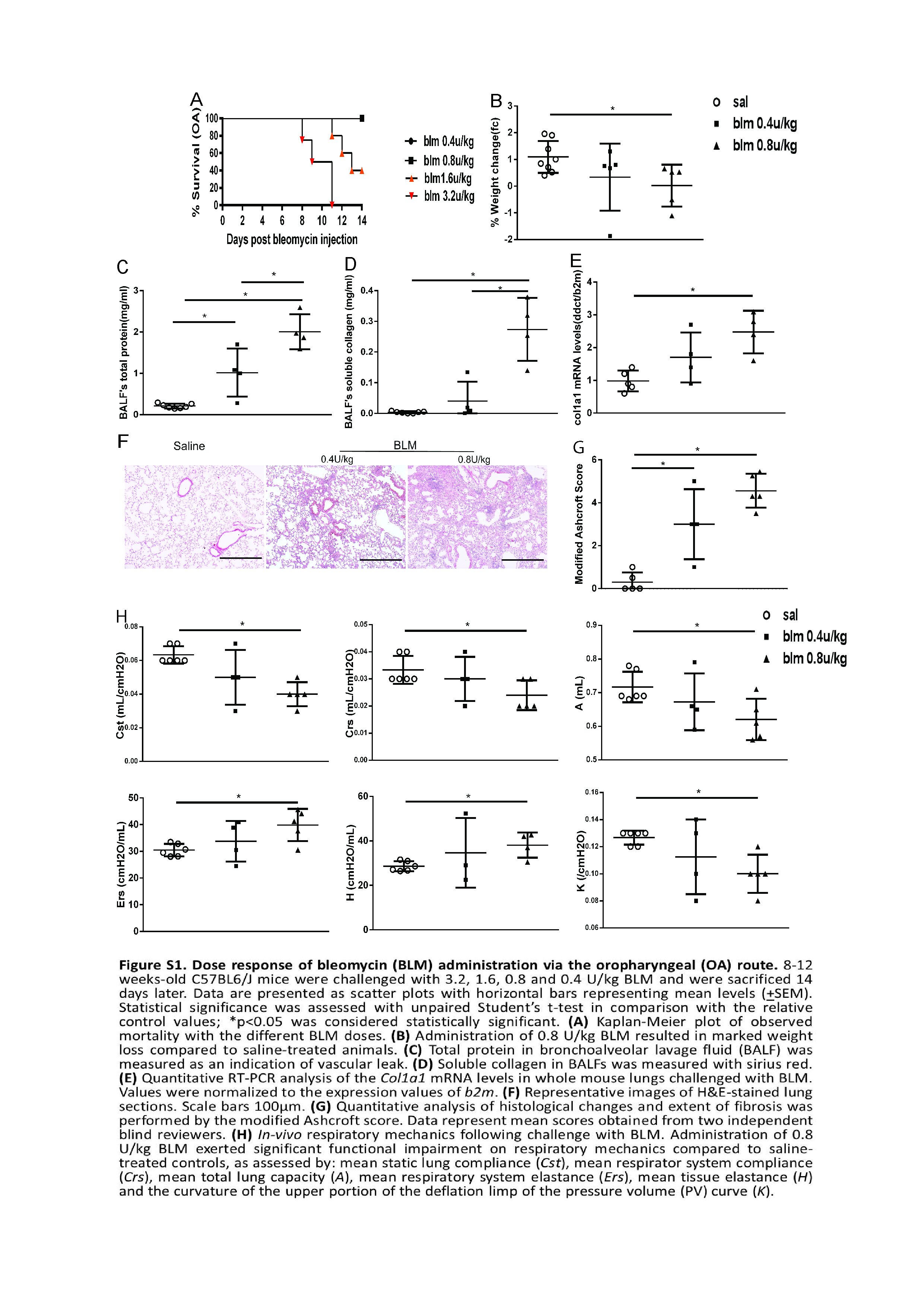

Supplement: Supplementary file 3 [file Image_1.TIF]

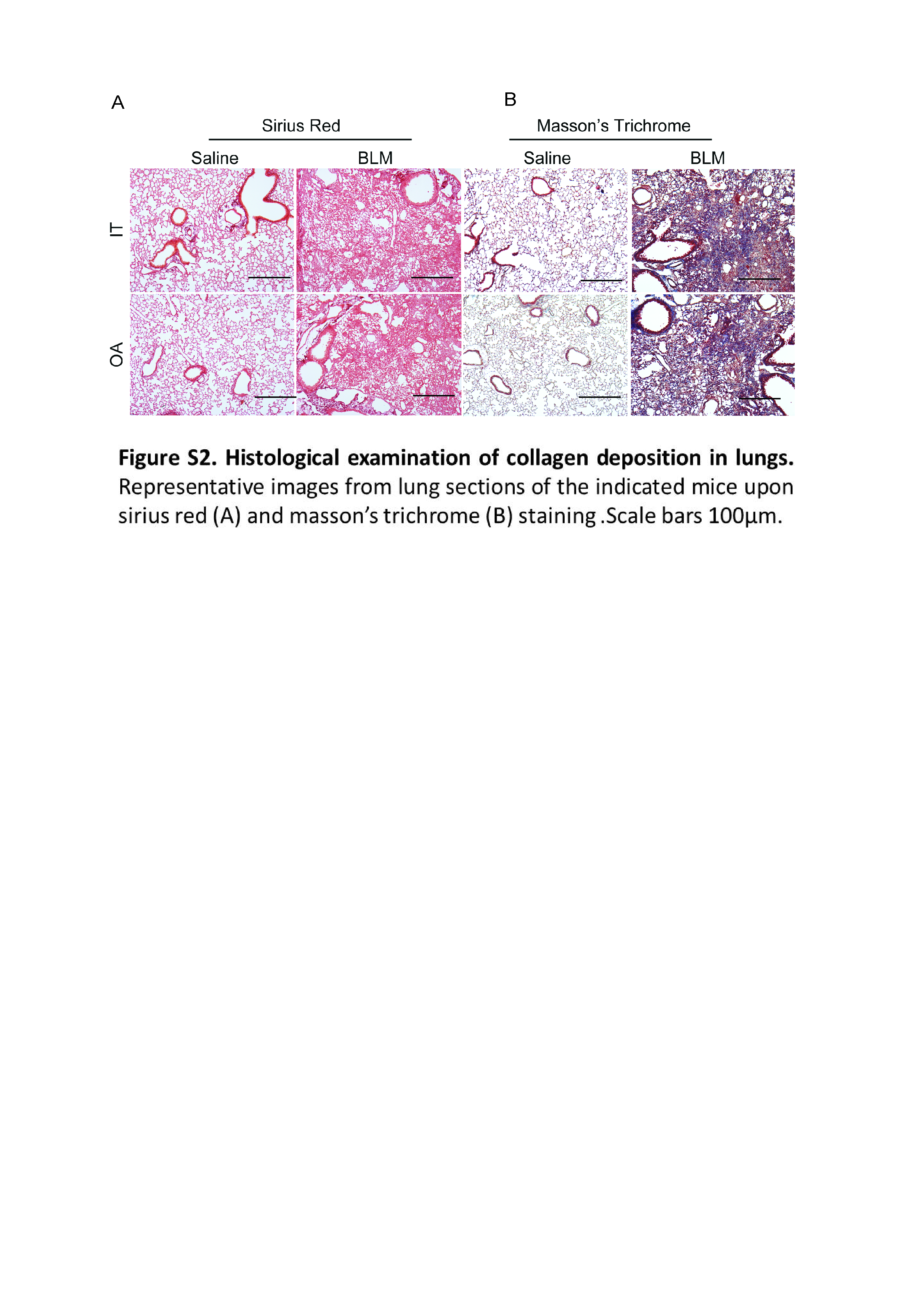

Supplement: Supplementary file 4 [file Image_2.TIF]
